# Virtual reality exposure and response prevention in the treatment of obsessive-compulsive disorder in patients with contamination subtype in comparison with in vivo exposure therapy: a randomized clinical controlled trial

**DOI:** 10.1186/s12888-022-04402-3

**Published:** 2022-11-28

**Authors:** Razieh Javaherirenani, Seyede Salehe Mortazavi, Mohammadreza Shalbafan, Ahmad Ashouri, Abbas Ramezani Farani

**Affiliations:** 1 Present Address: Present Address: Department of Clinical Psychology, School of Behavioral Sciences and Mental Health (Tehran Institute of Psychiatry), Iran University of Medical Sciences, Tehran, Iran; 2 Present Address: Present Address: Geriatric Mental Health Research Center, School of Behavioral Sciences and Mental Health (Tehran Institute of Psychiatry), Iran University of Medical Sciences, Tehran, Iran; 3 Present Address: Present Address: Mental Health Research Center, Psychosocial Health Research Institute. Department of Psychiatry, School of Medicine, Iran University of Medical Sciences, Tehran, Iran; 4 Brain and Cognition Clinic, Institute for Cognitive Sciences Studies, Tehran, Iran; 5 Department of Clinical Psychology, School of Behavioral Sciences and Mental Health (Tehran Institute of Psychiatry), Iran University of Medical Sciences, Tehran, Iran

**Keywords:** Contamination, Exposure and response prevention, Obsessive-compulsive disorder, Virtual reality

## Abstract

**Background:**

Obsessive-Compulsive Disorder (OCD) is characterized by disturbing and unwanted thoughts as well as repetitive and time-consuming behaviors that interfere with performance. Cognitive Behavior Therapy (CBT) has shown to have beneficial effects on reducing OCD symptoms as the first line of treatment. Moreover, Virtual Reality (VR) has been a more feasible and accessible intervention for OCD in recent years. Regarding the point, the objective of this study was to evaluate the effectiveness of virtual reality exposure and response prevention (VRERP) in the treatment of the OCD contamination subtype.

**Methods:**

A total number of 36 adults with OCD-contamination subtype were registered and randomly assigned to the intervention and control groups. The intervention group received a 60-min CBT including a “contaminated” virtual environment while the control group received CBT as a standardized treatment. Out of these, 29 patients completed the treatment in 12 weekly sessions. The patients completed the Yale-Brown Obsessive-Compulsive Scale (Y-BOCS), Beck Depression Inventory-II (BDI-II), Beck Anxiety Inventory (BAI), Obsessive Beliefs Questionnaire-44(OBQ-44), and World Health Organization Disability Assessment Scale-2 (WHODAS-2) at week 0, week 12 and after 3 months follow-up.

**Results:**

Based on the results of the repeated measure analysis of variances, the total score of obsession and compulsion subscales of Y-BOCS significantly decreased as a primary outcome in the intervention group (*F* = 60.97, *P* < 0.001, partial eta squared = 0.82; *F* = 20.46, *P* < 0.001, partial eta squared = 0.61; *F* = 29.57, P < 0.001, partial eta squared = 0.69; respectively). The total score of BDI-II and BAI was reduced in both groups but there was no significant difference between them (BDI-II: *F* = 0.54, *P* = 0.47, partial eta squared = 0.02; BAI: *F* = 3.12, *P* = 0.06, partial eta squared = 0.19). However, there was a significant difference in the OBQ-44 (*F* = 16.78, *P* < 0.001, partial eta squared = 0.56) and the total WHODAS-2 score between the groups (*F* = 14.64, *P* < 0.001, partial eta squared = 0.53).

**Conclusions:**

This study demonstrated the effectiveness of VRERP in the treatment of the OCD-contamination subtype. Therefore, VRERP can be used in CBT as an alternative exposure tool.

**Trial registration:**

Iranian Registry of Clinical Trials, IRCT ID: IRCT20210214050353N1, Registered on 16/10/2021.

**Supplementary Information:**

The online version contains supplementary material available at 10.1186/s12888-022-04402-3.

## Background

Obsessive-compulsive disorder (OCD) is characterized by persistent, annoying thoughts together with images or obsessions and /or repetitive behaviors or mental actions performed to decrease anxiety and distress [1]. Fear of contamination is the most prevalent symptom of OCD [2, 3]. Patients with OCD-contamination subtype overestimate the severity and likelihood of contamination. Contamination obsessions often lead to washing and cleaning compulsions [4] and compulsive rituals are performed to avoid contamination, germ, and dirt and reduce anxiety. In addition, they avoid feared situations and objects that provoke contamination-related obsessive thoughts [5].

Obsessive thoughts of contamination lead to anxiety and the person suffering from obsession begins to perform compulsive behaviors to reduce anxiety, and by doing these actions, anxiety temporarily reduced, but quickly new triggers of anxiety emerge and the cycle of obsession begins. But every time these actions are performed, the doubt related to decontamination and the feeling of inability to do it properly increases, and as a result, the patients avoid doing many daily actions related to the content of their obsession or feel disable against them. This inability continues to such an extent that it leads to learned helplessness and the patients suffer from depression due to this failure.

So**,** considering the relationship between the severity of OCD symptoms and performance deficits, it is important to investigate whether treatment improvement from OCD leads to improvement in performance and ability, but few studies have investigated the relationship between the improvement of OCD symptoms and the performance of the people with this disorder after receiving treatment [6]. In addition, this evidence emphasizes the importance of measuring anxiety and depression when evaluating therapeutic changes in OCD and examining the role of these disorders, which may independently lead to simultaneous improvements in OCD symptoms [7].

The most common treatment for patients with OCD is selective serotonin reuptake inhibitors (SSRIs) and cognitive-behavior therapy (CBT), which are used separately or together [8]. Selective serotonin reuptake inhibitors reduce the OCD symptoms in 40 to 60% of the patients [9]. Cognitive-behavior therapy, especially exposure and response prevention (ERP) is also effective [10] but 30% of patients did not show any improvement in their symptoms.

In cognitive-behavior therapy, patients are exposed to anxious stimuli but they do not respond to the obsessions. At first, the therapist explains OCD and its treatment. Then, external and internal stimuli are identified that provoke obsessive thoughts and subsequent distress. They identify the feared outcome if the rituals are not performed. Then, they develop an anxiety hierarchy according to different situations from the least to the most distressing. While exposed to distressing stimuli, the therapist gives some instructions to guide the patient to prevent compulsions and how to tolerate the distress. In imaginal exposure, patients visualize their feared outcome provoked by their obsessive thoughts. Thus, patients learn that the feared consequences do not happen, and that they can tolerate anxiety without compulsion. Then, the therapist and the patient discuss the patient’s experience and learning during in vivo and imaginal exposure [11].

Although 33% of the patients cannot imagine the situations in imaginal exposure [12] and use cognitive avoidance during this exposure [13]. In addition, the patients should expose to very anxious stimuli in real situations in vivo exposure that lead to a high dropout rate in the early treatment [14]. There is much evidence that exposure and response prevention (ERP) should be the first-line treatment for OCD [15]. Patients are exposed to feared and anxious objects or stimuli in ERP without performing compulsions. The patients are confronted with real phobic stimuli/situations (in vivo exposure), whereas they visualizes a feared situation in their mind in imaginal exposure. Creating real objects or situations in vivo exposure is difficult, costly, and sometimes impossible [16]; therefore, many patients reject ERP [15]. Imaginal exposure helps patients confront anxious stimuli without safety problems, such as the real risk of contamination but it is not real and fails to provoke anxiety [16]. Thus, a new method is needed to overcome the current limitations in exposure therapy.

Virtual reality (VR) is a computer-generated simulation where users interact with the environment in real-time [17]. VR provides a supportive and secure environment for the patient and increases self-efficacy [18, 19], so VR exposure is more acceptable than in vivo or imaginal exposure [20, 21]. The development of VR has increased the use of VRET for patients who do not want to expose to much-feared situations since VR can provide step-by-step exposure to feared stimuli in a more realistic and controlled way in a safe setting. Also, VR can overcome the limitations of traditional ERP techniques through the specific capabilities it posseses. VR provides flexibility (adapting virtual scenarios to the patients’ needs and fears), reproducibility (each scenario can be repeated and executed exactly), controlled nature (adjusting the level of anxiety in the stimuli), safety (every situation can finish without aversive results), and confidentiality (supportive and private environment without feeling embarrassment) [22].

Finally, this study, with the help of virtual reality technology, tries to offer a coherent and complete CBT by observing the points mentioned in the treatment protocols, including setting goals, psychological training of the principles by the therapist, determining assignments, evaluating the treatment process through questionnaires and so on. In this research, in order to improve the ecological credibility and immersion of the virtual reality environment, the first person perspective was used to create meaningful scenarios. Also, unlike previous studies, the sample size of this study was larger and CBT was used as a standard treatment for the control group. Also, the comparative results of two treatments were followed up for 3 months whereas the previous studies were mostly pilot studies without a control group and had a small sample size [4, 13, 23–26].

Generally, research show that VR exposure is an appropriate alternative for the treatment of OCD, however, there has been limited research on the effect of VR exposure on the fear of contamination. In addition, no research has been done on the effect VR exposure on contamination in OCD in Iran. Thus, this study aimed to explore whether contamination-related VR scenarios can reduce the severity of OCD symptoms, depression, anxiety, obsessional thoughts and disability.

## Methods

### Trial setting and design

A 12-week, randomized, single-blinded, parallel-group trial was performed at the outpatient clinics of Tehran Institute of Psychiatry (affiliated with Iran University of Medical Sciences, Tehran, Iran) from October 2021 to January 2022. Randomization process was done by Excel 2007 using random block sizes of 4 resulting in 18 participants in each group. The random allocation sequence was done by one co-researcher who was not involved in the trial. However, a research assistant, who registered the participants and assigned them to the intervention and the control groups, was aware of the allocation sequence. The allocation sequence were concealed using sequentially numbered, sealed, opaque envelopes. Regarding to the nature of the study, the patients and the clinician were not blinded whereas outcome assessors and data analysts were blinded to the allocation.

To design virtual scenarios, studies conducted on virtual reality treatment for OCD especially contamination subtype were reviewed and since the scenarios could not be used considering Iranian culture, some culture-dependent scenarios were developed in two steps. In the first step, the content of the scenarios was determined and approved by one psychiatrist and three clinical psychologists according to the objectives of the study and in the second step, the scenarios were developed. The VR environments resembled the average Iranian home and were designed to induce contamination impulses.

### Participants

**Fig. 1:**
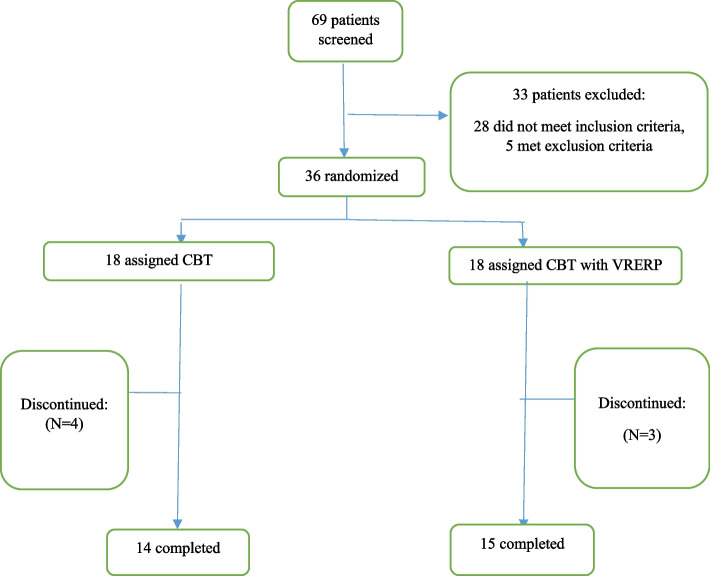
Trial/Participants Flow-diagram

Sixty-nine patients aged 18-50 years, with a clinical diagnosis of OCD based on the Diagnostic and Statistical Manual of Mental Disorders, 5th Edition (DSM-5) criteria were primarily screened but 36 patients were recruited and randomly assigned to the intervention (*N* = 18) and the control (*N* = 18) groups; out of which, 29 patients completed the study (Table 1). All of the patients enrolled in the study were assessed considering DSM-5 through a structured clinical interview by a psychiatrist for the inclusion criteria [27]. Following their informed consent, each participant was clinically interviewed to assess whether the candidate’s symptoms were consistent with OCD and whether the current primary obsession was contamination, and to characterize OCD symptoms. The exclusion criteria included: 1) having a current or history of auditory or visual impairment; 2) traumatic brain injury; 3) severe neurologic illness; 4) severe personality and psychotic disorders; 5) substance abuse or dependence; and 6) bipolar disorder. During the trial, the patients were not allowed to receive any parallel psychotherapy. Also, the dosage and type of medication did not change. The trial flow diagram and number of dropouts are represented in Fig. 1.

**Table 1:** Demographic characteristics of the participants

Demographic variables		Control group		Intervention group	
		Mean ± SD	Number (%)	Mean ± SD	Number (%)
Age (years)		38 ± 9.703	14(48.27)	35.666 ± 7.518	15(51.72)
Sex	Male		6 (42.9)		1 (6.7)
	Female		8 (57.1)		14 (93.3)
Marital status	Single		5 (35.7)		6 (40)
	Married		9 (64.3)		9 (60)
Education	Diploma		7 (50)		8 (53.3)
	Bachelor		5 (35.7)		6 (40)
	Master		1 (7.1)		1 (6.7)
	Ph.D.		1 (7.1)		0 (0)

### Interventions

Eligible participants were randomly assigned to the intervention or control groups for 12 weeks. Cognitive-behavioral therapy was performed according to Leahy and Hollands’ treatment plans and interventions for OCD [28]. The participants completed several self-report measures such as the Yale-Brown Obsessive-Compulsive Scale, Beck Depression Inventory-II, Beck Anxiety Inventory, Obsessive beliefs Questionnaire-44, and World Health Organization Disability Assessment Scale-2 in week 0, week 12, and 3 months after the end of the treatment. The first three sessions were dedicated to case formulation, introducing treatment plans and OCD, collecting information about obsessive fears and rituals, and developing an exposure hierarchy of anxiety-provoking situations.

The first exposure sessions were related to mild anxiety-provoking situations and gradually progressed to situations that caused more anxiety. At each stage, the patients expressed the level of anxiety on a scale from 0 to 100 after every 5 minutes. When the patients’ subjective evaluation of the anxiety decreased, and they could tolerate the anxiety well at that stage, they shift to the next most anxiogenic scenario. Each session began with a review of the material of the previous session, homework, self-monitoring of obsessions and compulsion, and cognitive challenging of dysfunctional thoughts. The content of sessions in both groups are presented in Tables 2 and 3.

**Table 2:** The content of sessions in the intervention group [28]

Sessions	Sessions Content
1	The evaluation of obsessions and compulsions, internal and external triggers of anxiety related to OCD, psychoeducation about OCD and CBT, and goal setting. Homework: Reviewing the therapeutic goals, changing or adding them if necessary.
2	The evaluation of motivation and monitoring the previous session homework, psychoeducation about the difference between obsessions and compulsions, explaining the cognitive behavior, conceptualization of OCD and the rationale of VRERP. Homework: Evaluation of obsessions and compulsions, self-monitoring, making a list of all rituals.
3	The evaluation of motivation and monitoring the previous session homework, psychoeducation about negative automatic thoughts (NAT) and their evaluation, anxiety hierarchy and preparing it. Homework: Challenging with NAT.
4	Monitoring the previous session homework, challenging with NAT related to overestimation of threat and magical thoughts, VRERP according to hierarchy, discussing about the patient’s experience and learning during the exposure. Homework: Exposure to feared stimuli in daily life according to in-session exposure, challenging with NAT related to overestimation of threat and magical thoughts, practicing to stop rituals.
5	Monitoring the previous session homework, examining the exposure process during the week and obstacles, answering the patients’ questions, reviewing the progress of goals, challenging with NAT related to responsibility and vulnerability to injury, VRERP according to higher hierarchy, discussing the patient’s experience and learning during the VR exposure. Homework: Repeating daily exposure to new situations according to hierarchy, challenging with NAT related to responsibility and vulnerability to injury, practicing to stop rituals.
6	Monitoring the previous session homework, examining the exposure process during the week, monitoring safety seeking and avoidance behaviors, challenging with thoughts related to avoidances and rituals, VRERP according to higher hierarchy, discussing about the patient’s experience and learning during the VR exposure. Homework: Exposure during the week, challenging with thoughts related to avoidances and rituals, practicing to stop rituals.
7	Monitoring the previous session homework, examining the exposure process during the week and obstacles, answering the patients’ questions, reviewing the progress of goals, educating about cognitive techniques such as examining the evidence and pie chart, VRERP to higher hierarchy, discussing the patient’s experience and learning during the VR exposure. Homework: Exposure during the week, challenging with NAT by examining the evidence and pie chart.
8	Monitoring the previous session homework, examining the exposure process during the week and obstacles, answering the patients’ questions, reviewing the progress of goals, cognitive technique about the difference between possibility and probability, VRERP to higher hierarchy, and discussing the patient’s experience and learning during the VR exposure. Homework: Exposure during the week, challenging with NAT by learned techniques.
9	Monitoring the previous session homework, examining the exposure process during the week and obstacles, answering the patients’ questions, reviewing the progress of goals, reviewing progress in identifying and correcting NAT, VRERP to higher hierarchy, discussing the patient’s experience and learnings during the VR exposure, encouraging the patient to use cognitive skills to deal with stressors. Homework: Exposure during the week, challenging with NAT by learned techniques.
10	Monitoring the previous session homework, examining the exposure process during the week and obstacles, answering to the patients’ questions, and reviewing the progress of goals, VRERP to higher hierarchy, discussing about the patient’s experience and learning during the VR exposure. Homework: Exposure during the week, challenging with NAT by learned techniques.
11	Monitoring the previous session homework, examining the exposure process during the week and obstacles, answering the patients’ questions, reviewing the progress of goals, VRERP to the highest hierarchy, discussing the patient’s experience and learning during the VR exposure, preparing the patient to termination, reviewing the learned techniques, evaluating next possible stressors and coping skills to overcome stressors, Homework: Exposure during the week, challenging with NAT by learned techniques.
12	Reviewing the therapeutic goals, education about lapse and relapse, relapsing prevention strategies, reviewing all treatment sessions and changes, encouraging the patient to exposure and plan for it, and to use learned techniques. Homework: Encouraging the patient to use all the learned skills and techniques.

**Table 3:** The content of sessions in the control group [28]

Sessions	Sessions content
1	The evaluation of obsessions and compulsions, internal and external triggers of anxiety related to OCD, psychoeducation about OCD and CBT, and goal setting. Homework: Reviewing the therapeutic goals, changing or adding them if necessary.
2	The evaluation of motivation and monitoring the previous session homework, psychoeducation about the difference between obsessions and compulsions, explaining the cognitive behavior, conceptualization of OCD and the rationale of ERP. Homework: Evaluation of obsessions and compulsions, self-monitoring, making a list of all rituals.
3	The evaluation of motivation and monitoring the previous session homework, psychoeducation about negative automatic thoughts (NAT) and their evaluation, anxiety hierarchy and preparing it. Homework: Challenging with NAT.
4	Monitoring the previous session homework, challenging with NAT related to overestimation of threat and magical thoughts, ERP according to the hierarchy, discussing about the patient’s experience and learning during the exposure. Homework: Exposure to fearful stimuli in daily life according to in-session exposure, challenging with NAT related to overestimation of threat and magical thoughts, practicing to stop rituals.
5	Monitoring the previous session homework, examining the exposure process during the week and obstacles, answering the patients’ questions, reviewing the progress of goals, challenging with NAT related to responsibility and vulnerability to injury, ERP according to higher hierarchy, discussing the patient’s experience and learning during the exposure. Homework: Repeating daily exposure to new situations according to hierarchy, challenging with NAT related to responsibility and vulnerability to injury, practicing to stop rituals.
6	Monitoring the previous session homework, examining the exposure process during the week, monitoring safety-seeking and avoidance behaviors, challenging with thoughts related to avoidances and rituals, ERP according to higher hierarchy, and discussing about the patient’s experience and learning during the exposure. Homework: Exposure during the week, challenging with thoughts related to avoidances and rituals, practicing to stop rituals.
7	Monitoring the previous session homework, examining the exposure process during the week and obstacles, answering the patients’ questions, reviewing the progress of goals, educating about cognitive techniques such as examining the evidence and pie chart, ERP to higher hierarchy, discussing the patient’s experience and learning during the exposure. Homework: Exposure during the week, challenging with NAT by examining the evidence and pie chart.
8	Monitoring the previous session homework, examining the exposure process during the week and obstacles, answering the patients’ questions, reviewing the progress of goals, cognitive technique about the difference between possibility and probability, ERP to higher hierarchy, discussing the patient’s experience and learning during the exposure. Homework: Exposure during the week, challenging with NAT by learned techniques.
9	Monitoring the previous session homework, examining the exposure process during the week and obstacles, answering the patients’ questions, reviewing the progress of goals, reviewing progress in identifying and correcting NAT, ERP to higher hierarchy, discussing the patient’s experience and learnings during the exposure, encouraging the patient to use cognitive skills to deal with stressors. Homework: Exposure during the week, challenging with NAT by learned techniques.
10	Monitoring the previous session homework, examining the exposure process during the week and obstacles, answering to the patients’ questions, and reviewing the progress of goals, ERP to higher hierarchy, discussing about the patient’s experience and learning during the exposure. Homework: Exposure during the week, challenging with NAT by learned techniques.
11	Monitoring the previous session homework, examining the exposure process during the week and obstacles, answering the patients’ questions, reviewing the progress of goals, ERP to the highest hierarchy, discussing the patient’s experience and learning during the exposure, preparing the patient for termination, reviewing the learned techniques, evaluating next possible stressors and coping skills to overcome stressors, Homework: Exposure during the week, challenging with NAT by learned techniques.
12	Reviewing the therapeutic goals, education about lapse and relapse, relapsing preventions strategies, reviewing all treatment sessions and changes, encouraging the patient to exposure and plan for it, and to use learned techniques. Homework: Encouraging the patient to use all the learned skills and techniques.

In the intervention group, VR equipment was used for exposure including software and hardware. Considering the hardware, a personal desktop computer and a color head-mounted display (HMD) with a 3-degrees-of-freedom tracker was used. VR environment was designed by Pishgaman Vagheiat Majazi Iranian (Ravana) Company (https://ravanavr.com/). The screen view was from a first-person perspective. In the software, each part of the home was designed in a way to represent a degree of contamination different from the other parts to give the therapist and the patient the freedom to gradually (not suddenly) be exposed to the contamination.

In the virtual reality program of the current research, two environments with moderate and severe dirt were designed. The participants used a joystick and tracker to move through the virtual environment, which included the corridor, the reception room, the kitchen, the bathroom and the toilet. By clicking the start button, the participants entered the virtual environment and during the exposure, they expressed their subjective units of distress scale (SUDS) using the range of 1 to 10. In the first session, in order to get familiar with the tools and equipment of the virtual environment, after entering the environment, the participants received instructions such as walking around the house, entering the reception, walking in the kitchen and reception and reaching to the bathroom door, opening the bathroom door and entering it.

In subsequent sessions, the participants were gradually exposed to fearful stimuli according to the designed hierarchy. Different parts of the house included objects that triggered obsessive thoughts of contamination. In each environment, there were all kinds of interactive objects that the participants had to look at closely, move them, touch them with a virtual hand, or put them together. For example, in the virtual environment of the kitchen, in the initial sessions, the patients touched objects that had the least amount of contamination, such as containers in the refrigerator, oven, kitchen cabinets, the floor and the walls of the kitchen. In the middle sessions, they encountered and touched objects that had an average level of contamination, including eating utensils, knives, spoons, and forks.

In the last sessions, they touched objects having a higher level of contamination, such as dustbin, rotten fruits in containers, etc. Each task was repeated and continued until the level of anxiety decreased or disappeared. In the virtual environment, the participants were not able to perform compulsive behaviors and could not get rid of the dirt and contamination. For example, water did not come out of the kitchen faucet. By clicking the end button, the participants left the virtual reality environment. The weekly exposure sessions lasted about 25 to 45 minutes. Between sessions, the patients were encouraged to expose anxiety-provoking stimuli and situations at least once a day, to reproduce the actions experienced in the virtual environment in real life, and to gradually eliminate rituals and compulsive behaviors in their daily life.

### Outcome measures

Several instruments were used considering the objectives of the study. The first instrument of the study was Y-BOCS used to assess the severity of obsessive-compulsive symptoms [29–31] of the patients at week 0, week 12, and after 3 months follow-up. This scale includes 10 items that are rated from 0 (no symptoms) to 4 (extreme symptoms) [32]. The psychometric properties of the Persian version of Y-BOCS were approved in the previous studies and optimal levels of internal consistency scores (symptom checklist 0.97, severity scale 0.95), split-half reliability (symptom checklist 0.93, severity scale 0.89), and test-retest reliability (0.99) were estimated [33]. The total score of the Y-BOCS difference between week 0, week 12, and 3 months follow-up was the primary outcome measure of the trial.

The second outcome measure was BDI-II used to assess the severity of depression symptoms. BDI-II is a 21-question multiple-choice self-report inventory in which high total scores indicate more severe depressive symptoms (0–13: minimal, 14-19: mild, 20-28: moderate, 29-63: severe) [34]. BDI-II-Persian version has a high internal consistency (*α* = .87) and test-retest reliability (*r* = 0.74) [35].

Also, BAI was applied to assess the severity of anxiety symptoms. The BAI consists of 21 questions which are rated from 0 (not at all) to 3 (severely) in which higher total scores indicate more severe anxiety symptoms (0–7: minimal, 8-15: mild, 16-25: moderate, 26-63: severe) [36].

Another instrument was the Obsessive Beliefs Questionnaire (OBQ-44) which was used to assess beliefs which are important in the development and maintenance of OCD. OBQ-44 is rated 1(completely disagree) to 7(completely agree) [37].

Moreover, WHODAS-2 was used to assess disability in 6 areas. This scale has 36 items which are rated from (1: without disability, 2: minimal disability, 3: moderate disability, 4: severe disability, and 5: extremely severe disability) [38]. The Persian version of WHODAS-2 demonstrated the appropriate internal consistency (78%) and Cronbach’s alpha (95%) [39].

Finally, There was no difference in characteristics between the two groups and the analysis showed that there was no statistically significant difference between the two groups (Table 4).

**Table 4:** Main outcome baseline data comparison

Measure	Group	N	Mean	Std. Deviation	*p*-value
YBOCS – week 0	control	14	28.8571	7.26273	0.21
	intervention	15	31.6000	3.83219	
OBSE– week 0	control	14	15.0714	3.49647	0.30
	intervention	15	16.2000	2.14476	
COMP-week 0	control	14	13.7857	4.35322	0.22
	intervention	15	15.4000	2.50143	
BDI– week 0	control	14	19.8571	3.43863	0.26
	intervention	15	31.1333	36.62721	
BAI– week 0	control	14	37.9286	15.13765	0.78
	intervention	15	39.2667	10.55236	
OBQ– week 0	control	14	178.3571	30.11416	0.57
	intervention	15	184.1333	25.17898	
DAS– week 0	control	14	103.6429	28.48915	0.48
	intervention	15	111.8000	32.50978	

### Sample size and statistical analyses

G-Power software version 3.1.9.2 [40] was used to compare two independent means and 28 participants were calculated as the sample size (14 in each group). Considering a drop-out rate of 30%, medium effect size (*d* = 0.25), beta 0.2, and alpha 0.5, the sample size was calculated 36 (18 in each group). All statistical analyses were done using SPSS version 18. Comparison of Y-BOCS scores as the primary outcome in the intervention and control groups in each assessment (week 0, week 12, and 3 months follow-up) was made using the repeated measure analysis of variances. Continuous variables were reported by mean ± SD and categorical variables by number (%). A p-value of less than 0.05 was considered to be significant.

### Adverse effects

Previous findings showed that low-weight HMD and high-quality image and resolution can reduce negative effects in the use of VR [41]. Also, repeated exposure can result in habituation and the adverse effects reduce between 1 to 7 days due to the adaptation to the virtual environment [42] and [43]. Some guidelines are used in this trial to minimize the side effects in the VR setting [44]. These included:

- Waiting 15 min after any exposure and not carrying out sensitive tasks such as driving, crossing the street, and going up and down the stairs.

- Informing the participants about the adverse effects of VR to reduce anxiety and fear about this new experience.

- Providing complete training about the use of input devices and coaching the patients.

- Providing the confidence that the participant could terminate the exposure at any time.

- Monitoring patients about some signs and behaviors during VR exposure such as sweating, pallor, fidgeting with HMD, looking away from the display, and closing eyes.

Observing these guidelines made the side effects mild but two participants did not complete the treatment in the intervention group because they experienced nausea, headache, and dizziness.

## Results

### Y-BOCS, Y-BOCS obsession subscale, Y-BOCS compulsion subscale, BDI-II, BAI, OBQ-44 and WHODAS-2 total score trend for each group during the trial course

**Fig. 2:**
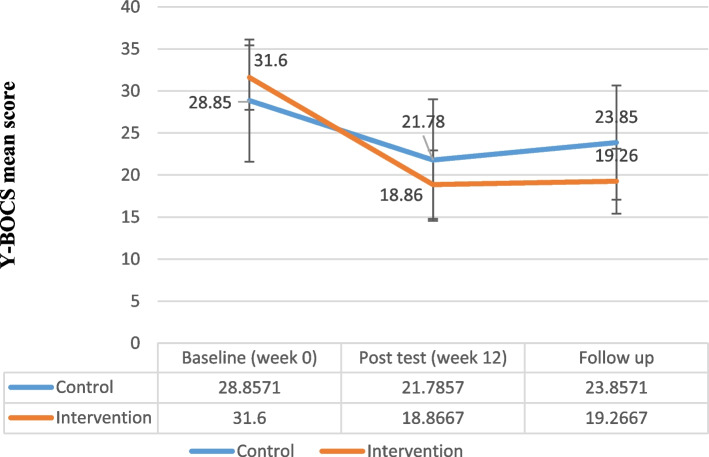
Yale-Brown Obsessive-Compulsive Scale (Y-BOCS) Total Score Trend for Each Group during the Trial

The results of repeated measure analysis of variances indicated that the total Y-BOCS score was significantly different between the groups (*F* = 60.97, *P* < 0.001, partial eta squared = 0.82) (Fig. 2, Table 5). Also, the total Y-BOCS obsession subscale score was significantly different between the two groups (*F* = 20.46, *P* < 0.001, partial eta squared = 0.61). Moreover, there was a significant difference between the intervention and the control groups in total Y-BOCS compulsion subscale score (*F* = 29.57, *P* < 0.001, partial eta squared = 0.69).

**Table 5:** Comparison of Y-BOCS, BDI-II, BAI, OBQ-44 and WHODAS-2 score change from week 0 in both groups

Control group			Intervention group			
Measure	Phase	Mean ± SD	Mean ± SD	Partial Eta Squared	P-Value	F
Y-BOCS	Week 0	28.857 ± 7.262	31.600 ± 3.832	0.82*	< 0.001	60.97
	Week 12	21.785 ± 7.223	18.866 ± 4.068			
	3 months Follow-up	23.857 ± 6.780	19.266 ± 3.863			
Compulsion subscale	Week 0	13.785 ± 4.353	15.400 ± 2.501	0.69*	< 0.001	29.57
	Week 12	10.428 ± 4.669	9.266 ± 2.086			
	3 months Follow-up	11.357 ± 4.307	9.600 ± 2.229			
Obsession subscale	Week 0	15.071 ± 3.496	16.200 ± 2.144	0.61*	< 0.001	20.46
	Week 12	11.357 ± 3.053	9.600 ± 2.557			
	3 months Follow-up	12.500 ± 3.228	9.666 ± 2.160			
BDI-II	Week 0	19.857 ± 3.438	31.133 ± 36.627	0.02	0.47	0.54
	Week 12	16.714 ± 3.625	25.200 ± 24.442			
	3 months Follow-up	16.928 ± 3.832	25.933 ± 23.398			
BAI	Week 0	37.928 ± 15.137	39.266 ± 10.552	0.19	0.06	3.12
	Week 12	28.285 ± 13.974	26.533 ± 5.998			
	3 months Follow-up	29.357 ± 13.859	26.466 ± 5.853			
OBQ-44	Week 0	178.357 ± 30.114	184.133 ± 25.178	0.56*	< 0.001	16.78
	Week 12	141.571 ± 29.016	130.266 ± 19.199			
	3 months Follow-up	141.428 ± 27.968	129.066 ± 19.177			
WHODAS-2	Week 0	103.642 ± 28.489	111.800 ± 32.509	0.53*	< 0.001	14.64
	Week 12	88.500 ± 27.317	89.866 ± 30.828			
	3 months Follow-up	93.428 ± 26.920	90.466 ± 30.220			

**Fig. 3:**
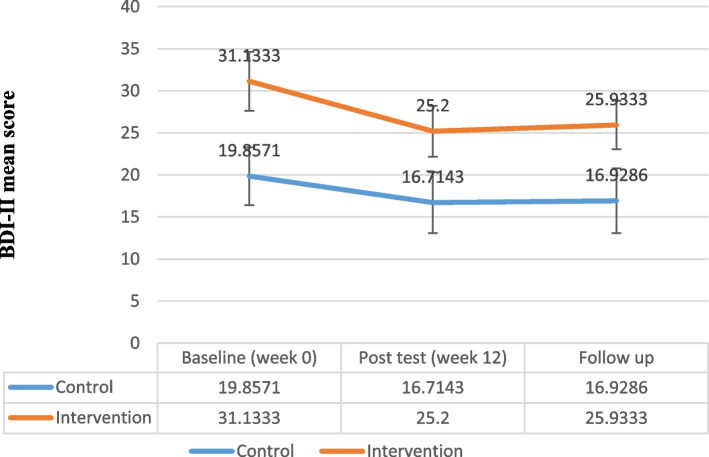
Beck Depression Inventory–II (BDI-II) Total Score Trend for Each Group during the Trial

**Fig. 4:**
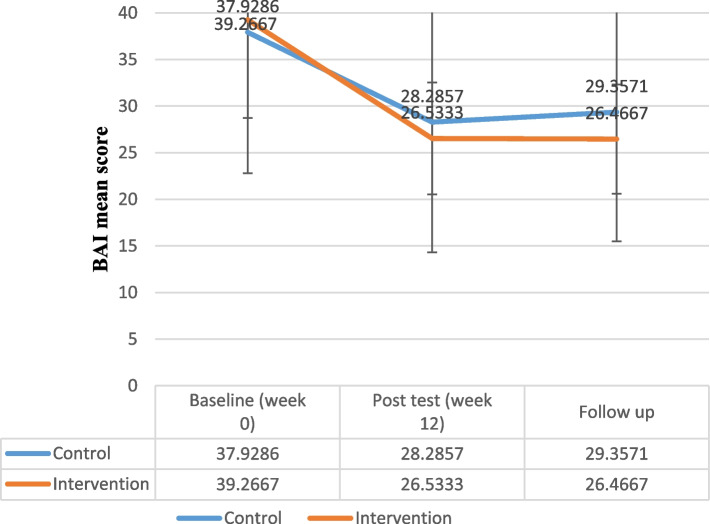
Beck Anxiety Inventory (BAI) Total Score Trend for Each Group during the Trial

In the follow-up, the total score of Y-BOCS had less return to the week 0 score in both groups. The findings of repeated measure analysis of variances revealed no significant difference in the BDI-II between the intervention and control groups (*F* = 0.54, *P* = 0.47, partial eta squared = 0.02) (Fig. 3, Table 5). In the follow-up, the total score of BDI-II had less return to the week 0 score in both groups. Also, there was no significant difference in total BAI score between the groups regarding the results of repeated measure analysis of variances (*F* = 3.12, *P* = 0.06, partial eta squared = 0.19) (Fig. 4, Table 5). In the follow-up, the total score of BAI did not return to week 0 and therapeutic gains were maintained 3 months after the treatment in the intervention group.

**Fig. 5:**
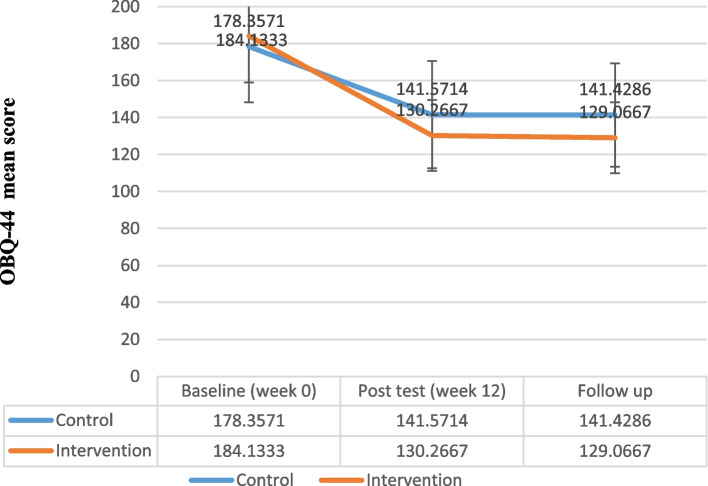
Obsessive Beliefs Questionnaire-44(OBQ-44) Total Score Trend for Each Group during the Trial

**Fig. 6:**
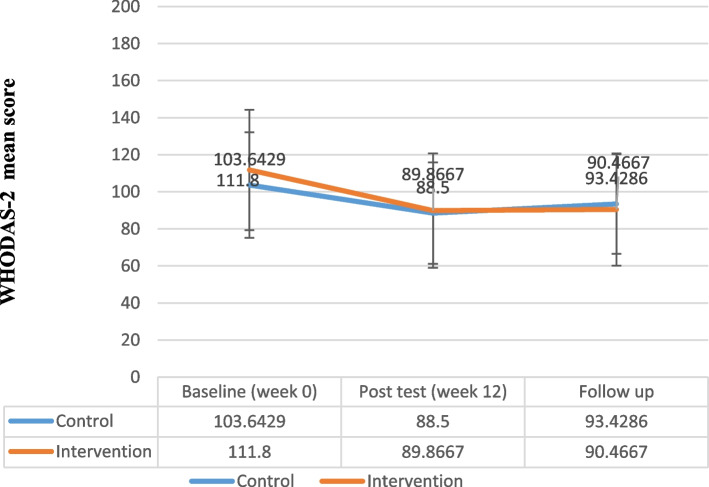
World Health Organization Disability Assessment Scale-2 (WHODAS-2) Total Score Trend for Each Group during the Trial

The results of repeated measure analysis of variances showed a significant difference in the OBQ-44 between the intervention and the control groups (*F* = 16.78, *P* < 0.001, partial eta squared = 0.56) (Fig. 5, Table 5). In the follow-up, the total score of OBQ-44 slightly decreased in the intervention group while it did not change in the control group. Finally, the findings of repeated measure analysis of variances revealed a significant difference in the WHODAS-2 score between the groups (*F* = 14.64, *P* < 0.001, partial eta squared = 0.53) (Fig. 6, Table 5).

The changes in outcome variables were statistically significant in all evaluated outcomes over time in the Table 6. Of course, the trend of these changes was not significantly different in the BDI-II in the two groups. The total score of Y-BOCS, Y-BOCS obsession and compulsion subscales, BAI, OBQ-44, WHODAS-2 has decresed with more slope over time in the intervent group but the trend of changes in BDI-II was not significantly different between two groups over time.

**Table 6:** Main effect (effect of time within groups) in Y-BOCS, BDI-II, BAI, OBQ-44 and WHODAS-2 scores

Measure	Partial Eta Squared	*P*-Value	F
Y-BOCS	0.97*	< 0.001	60.97
Compulsion subscale	0.95*	< 0.001	29.57
Obsession subscale	0.90*	< 0.001	20.46
BDI-II	0.72*	< 0.001	0.54
BAI	0.80*	< 0.001	3.12
OBQ-44	0.97*	< 0.001	16.78
WHODAS-2	0.53*	< 0.001	14.64

## Discussion

The objective of the present study was to evaluate the effectiveness of virtual reality exposure and response prevention (VRERP) in the treatment of OCD contamination subtype. The results of the study indicated that VR technology can be an effective treatment for OCD.

The findings revealed that CBT using VRERP significantly improved the severity of OCD symptoms in the intervention group compared to the control group. These results are in line with previous studies. For example, Laforest et al. [13] suggested that contaminated virtual environments can trigger and increase anxiety in OCD patients. Also, Laforest et al. [23] found that VRET can improve the usefulness of CBT for contamination-related OCD. Belloch et al. [26] used VR exposure on patients with contamination-based OCD and found that their anxiety levels increased as the level of dirtiness of the virtual environment increased. In addition, Inozu et al. [24] stated that repeated exposure to the VR environment can decrease the symptom severity of OCD, especially the contamination fear.

It is essential to mention that Immersion is one of the most important features of virtual reality, that is, the patient is immersed in the virtual world and can see all the details of the surrounding environment just like in the real world. The goal of virtual reality therapy is to provide deliberate and prolonged exposure of the patient to an anxiety-provoking situation and prevent the associated avoidance response. The patient experiences anxiety in the short term, but through the process of extinction in frequent exposure sessions, he or she experiences a reduction in anxiety and avoidance.

On the other hand, virtual reality causes step-by-step exposure to anxiety-provoking stimuli in a controlled and more realistic way than imaginal exposure as well as a more realistic and safer environment than in vivo exposure [25]. This reduces the patient’s distress and increases the success of the patient’s treatment. Instead of experiencing the difficulties of an in vivo or imaginal environment, patients enter the virtual world with more comfort and confidence and learn how to overcome their obsessions [45].

The therapist guides the participant in each moment and tells them what to do. Also, due to the flexibility of the virtual environment, the therapist can design the environment according to the patient’s needs, creating a sense of presence and interaction and better treatment results.

The current clinical trial did not demonstrate a significant difference in the improvement of depression symptom severity between the two groups. There was a slight reduction in the depressive symptoms which was consistent with the results of the study showing that changes in OCD symptoms did not significantly reduce depression symptoms [46].

Previous research has not presented conclusive results considering the effects of OCD treatment on reducing depression symptoms. Obsessive-compulsive disorder and depression are both separate disorders with different developmental histories as well as underlying factors. Also, the severity of depression symptoms, the number of years since the onset of the disorder, and its genetic background are involved in the recovery rate of patients’ depression symptoms.

The lack of difference between the two treatment groups in reducing the level of depression is mainly because the depression of these patients is secondary to their obsessive-compulsive disorder while the content of treatment sessions are mainly focused on reducing obsessive symptoms.

Furthermore, there was no significant difference in the reduction of anxiety symptoms between the groups. Some research indicates that virtual reality technology along with relaxation and anxiety management techniques plays a significant role in improving anxiety symptoms [47], while in the current research, virtual reality technology was not accompanied by the mentioned techniques and in the exposure sessions, the patient was confronted with anxiety-provoking objects and situations. Also, in obsessive-compulsive disorder, stimulating anxiety and breaking the connection between anxiety and obsessive behaviors of patients is considered the main rule of exposure therapy. Therefore, a longer period of treatment is needed to reduce anxiety levels significantly [48].

Moreover, research results show that if people are faced with scenarios that are specific to their problems and complaints, a significant reduction in their anxiety level occurs, but it is difficult to measure this change by self-report tools because these tools measure the general anxiety [24]. As a result, the reduction of depression and anxiety was not significantly different between the two groups, so the reduction of obsessive behaviors and beliefs can be due to the effect of the intervention.

Nonetheless, the findings showed a significant difference in the reduction of obsessional thoughts in the intervention group compared to those of the control group. These findings suggest that frequent exposure to feared stimuli can change cognition, behavior, as well as physical and emotional responses. In fact, with VRET, the therapist can customize the virtual reality content to the patient’s fears and then, guide and support the patients while supervising them in the virtual environment. The patients are involved in the scenario and they can practice anxiety management skills in a safe virtual environment before being exposed to the feared stimuli in real situations. After each VR exposure, the therapist and patients discuss the SUDS scores and patient’s experiences in the virtual environment, such as what the patient learns, how their dysfunctional beliefs have been corrected, and how their expectations regarding the feared stimuli have been rejected [49, 50].

Also, the findings indicated that the intervention reduced the severity of disability in the intervention group more significantly than that in the control group. The results of this research were consistent with previous studies [51–53] showing that there is a relationship between the severity of OCD symptoms and the level of performance and ability of the individuals since patients with OCD lose about 2.5% of their life due to disability [54]. Therefore, improving OCD symptoms can lead to positive changes in the patient’s social and professional activities, increased self-efficacy, and reduced restrictions [55].

Virtual reality directs the patient’s attention and concentration to the virtual environment intended by the therapist by involving them in images, colors, sounds, touchpads, audio and visual screens as well as providing movement and real physical activity, and creates a sense of presence. Through the sense of presence, patients can easily experience anxiety in the virtual environment, and the responses needed to face the situation are created in the individuals, assisting them to have better and more efficient performance and mastery in social situations [4].

In addition to, by breaking the pattern of avoidance behavior and increasing the individual’s belief in their own abilities and competences, VRET increases the patient’s motivation to move forward in the exposure process and thus reduces the patient’s disability [56]. In fact, people with OCD underestimate their ability to cope with fear and anxiety. When they can endure anxiety-provoking situations without avoidance, their sense of self-efficacy and ability increases because they realize their abilities and capacities [57].

Finally, with the improvement of obsessive-compulsive disorder, positive changes occur in various aspects of a person’s life, including daily activities, social and occupational activities. Furthermore, the reduction of symptoms and subsequently, the reduction of avoidance associated with this disorder will remove the limitations caused by the disorder, including limited relationships or enjoyable social activities and increase professional activities.

### Strengths and limitations

This study was a randomized controlled trial with a clinical sample diagnosed with OCD contamination subtype that provided the promising results in favor of the effectiveness of the VRERP in the treatment OCD. The use of contaminated environment can enhance the intervention results through VR’s potential to provoke high anxiety in the participants as if they were in the really feared situations. The study findings can be used as an evidence-based intervention protocol to improve the literature and make a noticeable contribution to the treatment of OCD.

However, despite its advantages, this trial was not without limitations. A single blinded study has an intrinsic bias. Study participants expected a positive potential effect of the intervention. Participants who are favorable to VR or new therapeutic intervention are likely to be participating in the study, and the control group might be disappointed or slightly discouraged from undergoing treatment. In fact, control intervention of CBT is good but disappointing in patients with expectations of new therapy. This aspect might explain why the control group showed less efficacy than the intervention group.

In addition, symptom assessment tools were not objective and BDI, BAI, and YBOC were all questionnaires indeed. Using objective tools or blinded assessment clinicians, participants’ behavior in the virtual space could be measured for the assessment of OCD symptoms such as: How long did participants look at or look off the dirt or disorganized space (avoidance, compliance of ERP)? How anxious participants feel by measuring heart rate, respiratory rate or skin conduction test(anxiety)? How often did they try to get rid of dirt or the delay time to the virtual ritual (compliance of ERP)? How well did they perform a target task (simple control task, for example, searching for necessary item to clear a mission, solving a problem etc.).

The lack of how to get rid of dirts in the virtual environment would make a participant anxious. It is different that there is no mean, and there is a mean but they choose not to do. No means to get rid of dirt would have positive effects for the participant who bears anxiety well, and the experience of a small success would propagate into the in vivo exposure-response prevention in the homework. However, participants who could not bear anxiety without compulsive behavior options might drop out of the VR session instead of performing compulsive behavior. Failure of ERP would be allowed in the VR program. More people with OCD will participate in the intervention and become familiar with the VR-based ERP training and eventually get relieved.

In addition, most of the participants were female, so the results should be generalized with caution. Moreover, in the virtual scenarios, there was no odor contamination to trigger more anxiety. Another limitation was the simulator sickness including dizziness, nausea, headache, and eyestrain.

### Implications for practice and research

Application of diverse and personalized virtual environments simulated to the patient’s feared stimuli can improve the findings of the future studies. Experimenters prevent compulsive behavior from happening by chance. This is impossible in the session (in vivo ERP). If the VR program reinforces ERP behavior by providing a positive stimulus, compulsive behavior is omitted by chance (operant conditioning, variable ratio positive reinforcement). VR-based ERP contains more potential based on behavioral science, which is not applicable in conventional CBT-OCD.

finally, it is suggested to replicate the methodology with a more diverse sample in a double-blind, randomized trial. It would also be interesting to develop VR for other subtypes of OCD. Future research with VRERP can use some components such as odor to provoke more anxiety. Finally, other studies can be conducted with longer follow-up periods to better evaluate the maintenance of therapeutic gains. It is recommended to use VR in children and adolescents with OCD in the therapeutic setting since the sample was completely composed of adults.

## Conclusion

The results showed that VR can be an appropriate, new and promising tool in the treatment of this subtype and can improve the effectiveness of CBT. Following these considerations, our results support the potential of VR in the treatment of OCD. VRERP in the session may encourage participants to jump into the in vivo EPR in the real world by the forced success experience. It can be a esasy alternative of ERP exposure but there is no means to engage in compulsive behaviors. It is very interesting and promising because OCD is quite difficult to treat by pharmacotherapy or CBT compared to depression and anxiety disorders. Finally, it should be mentioned that although VR was effective in the assessment and treatment of OCD, its use is still limited and in the primary stage. Therefore, more research is needed to increase the evidence regarding the effectiveness of using this new technology in the therapeutic and clinical settings.

## Supplementary Information

**Additional file 1.**

## Data Availability

The dataset supporting the conclusions of this article is included within the article (and its additional file: supplemental file 1).

## Abbreviations

OCD: Obsessive-Compulsive Disorder
VR: Virtual Reality
VRERP: Virtual Reality Exposure and Response Prevention
CBT: Cognitive Behaviora Therapy
Y-BOCS: Yale-Brown Obsessive Compulsive Scale
BDI-II: Beck Depression Inventory–II
BAI: Beck Anxiety Inventory
OBQ-44: Obsessive Beliefs Questionnaire-44
WHODAS-2: World Health Organization Disability Assessment Scale-2
SSRIs: Selective Serotonin Reuptake Inhibitors
DSM-5: Diagnostic and Statistical Manual of Mental Disorders, 5th edition
NAT: Negative Automatic Thoughts
SD: Standard Deviation
HMD: Head-Mounted Display
SUDS: Subjective Units of Distress Scale
